# Persistent Vomiting and Epigastric Pain in an Adolescent: A Case of Superior Mesenteric Artery Syndrome Unmasked

**DOI:** 10.3390/reports9010020

**Published:** 2026-01-09

**Authors:** Maria Rogalidou, Georgios Papagiannis, Konstantina Dimakou, Paraskevi Galina, Stavroula-Zoe Siska, Alexandra Papadopoulou

**Affiliations:** 1 Division of Gastroenterology & Hepatology, First Department of Paediatrics, National and Kapodistrian University of Athens, ‘Agia Sophia’ Children’s Hospital, 11527 Athens, Greece; 2 First Department of Paediatrics, National and Kapodistrian University of Athens, ‘Agia Sophia’ Children’s Hospital, 11527 Athens, Greece; 3 Gastroenterology Department, “Agia Sofia” Children’s Hospital, 11527 Athens, Greece; 4 Radiology Department, “Agia Sofia” Children’s Hospital, 11527 Athens, Greece

**Keywords:** superior mesenteric artery syndrome, wilkie’s syndrome, adolescent, duodenal obstruction, weight loss, gastrointestinal symptoms, vomiting

## Abstract

**Background and Clinical Significance:** Superior mesenteric artery syndrome (SMAS) is a rare and often underdiagnosed cause of proximal intestinal obstruction, resulting from compression of the third portion of the duodenum between the SMA and the aorta. It typically occurs in individuals with significant weight loss due to mesenteric fat depletion. **Case** **Presentation:** We report the case of a 14.5-year-old female presented with a 6-day history of intractable vomiting and epigastric pain, on a background of intermittent vomiting over the preceding six months associated with a 7 kg unintentional weight loss, culminating in inability to tolerate oral intake. Her clinical course was complicated by a transient episode of blurred vision, numbness, and incoherent speech, initially suspected to be a neurological event. Extensive gastrointestinal and neurological investigations were inconclusive. Elevated fecal calprotectin levels raised suspicion for inflammatory bowel disease, given her family history, though endoscopy and histopathology were unremarkable. Advanced imaging ultimately demonstrated a markedly reduced aortomesenteric angle (6°) and distance (4 mm), confirming the diagnosis of SMAS. The patient was initially managed conservatively with total parenteral nutrition (TPN), achieving partial weight gain of 5 kg after 8 weeks of TPN. Due to persistent duodenal compression, surgical intervention was required. At 7-month follow-up, the patient remained symptom-free with restored nutritional status and a good weight gain. **Conclusions:** This case highlights the importance of considering SMAS in adolescents with chronic upper gastrointestinal symptoms and significant weight loss. Early recognition and appropriate imaging are essential to diagnosis, and timely surgical management can lead to excellent outcomes when conservative treatment is insufficient.

## 1. Introduction and Clinical Significance

Superior Mesenteric Artery Syndrome (SMAS), also known as Wilkie’s Syndrome, is a rare and potentially life-threatening condition caused by compression of the third portion of the duodenum between the superior mesenteric artery and the abdominal aorta. The syndrome has a reported prevalence of 0.013–0.3% in the general population based on radiologic and autopsy studies [1]. In healthy individuals, the normal aortomesenteric angle ranges from 38° to 65°, and the aortomesenteric distance is typically 10 to 28 mm. It occurs most often in adolescents and young adults, particularly during rapid growth spurts, and shows a female predominance (female-to-male ratio ~3:2–4:1) [1,2].

This condition leads to extrinsic compression leading to partial or complete duodenal obstruction. It often presents with non-specific gastrointestinal symptoms like postprandial epigastric pain, nausea, early satiety, and vomiting, which may mimic more common causes of abdominal pain, particularly in adolescents.

This case report presents a teenage patient with recurrent vomiting and epigastric pain, ultimately diagnosed with SMAS after a delay, highlighting the importance of considering this rare diagnosis in adolescents with persistent gastrointestinal symptoms especially after significant weight loss.

## 2. Case Presentation

A 14.5-year-old adolescent female was admitted to our hospital with a 6-day history of vomiting and epigastric pain. She had lost 7 kg over the preceding six months, prior to the onset of symptoms. She had been previously hospitalized for 4 days at two regional hospitals without improvement. On the day of her transfer, she experienced an acute episode characterized by blurred vision, numbness of the right upper extremity and lips, and incoherent speech.

Her past medical history was largely unremarkable. Lately she developed, mild dyspeptic symptoms including bloating, belching, and constipation. At age 12, she was hospitalized due to a prolonged episode of epigastric pain accompanied by multiple vomiting episodes lasting 18 days. A comprehensive workup at that time—including a gastrointestinal transit study, brain MRI, and upper endoscopy—revealed no significant abnormalities.

Her family history was notable for inflammatory bowel disease: her sister had ulcerative colitis and underwent colectomy at the age of 18, while her paternal grandmother also had ulcerative colitis and passed away at the age of 47. Her mother has a history of hypertension and migraine.

### 2.1. Physical Examination

On admission, the patient appeared in moderate general condition. Her weight was 46.5 kg (23rd percentile), height 169 cm (90th percentile), and body mass index (BMI) was 16.3 kg/m^2^ (5th percentile). Vital signs were within normal limits: blood pressure 125/74 mmHg, heart rate 76 beats per minute, and temperature 37.2 °C.

Physical examination revealed a mildly erythematous and coated tongue. Pulmonary auscultation demonstrated normal vesicular breath sounds bilaterally. Cardiac examination showed clear and rhythmic heart sounds (S1 and S2) with a grade I/VI systolic murmur. The abdomen was soft and non-distended, with mild tenderness in the epigastric region and positive bowel sounds (++). No organomegaly or palpable masses were detected. Secondary sexual characteristics were consistent with Tanner stage IV for both breast and pubic hair development. Neurological examination was unremarkable; the patient was fully alert, oriented, and scored 15/15 on the Glasgow Coma Scale (GCS). No focal neurological deficits were noted.

### 2.2. Laboratory and Initial Diagnostic Workup

Initial investigations were directed toward identifying potential gastrointestinal and neurological causes of the patient’s symptoms.

Screening for celiac disease was negative. Stool antigen testing for Helicobacter pylori, stool cultures, Clostridium difficile toxin, and stool parasitology were all negative. Fecal occult blood tests were negative on multiple occasions, with the exception of one positive result. A lactose tolerance test was positive, consistent with lactose intolerance. Fecal calprotectin levels were markedly elevated, measured at 660 μg/g and 363 μg/g on two separate occasions (normal <50 μg/g), suggesting the presence of intestinal inflammation. A neurological examination was unremarkable. Brain computed tomography (CT) and fundoscopy revealed no abnormalities. The acute episode of blurred vision, numbness, and slurred speech was interpreted as consistent with a complicated migraine. Abdominal and pelvic ultrasound showed no pathological findings. Upper and lower gastrointestinal endoscopy, including histologic evaluation, was unremarkable.

### 2.3. Advanced Imaging

**Figure 1:**
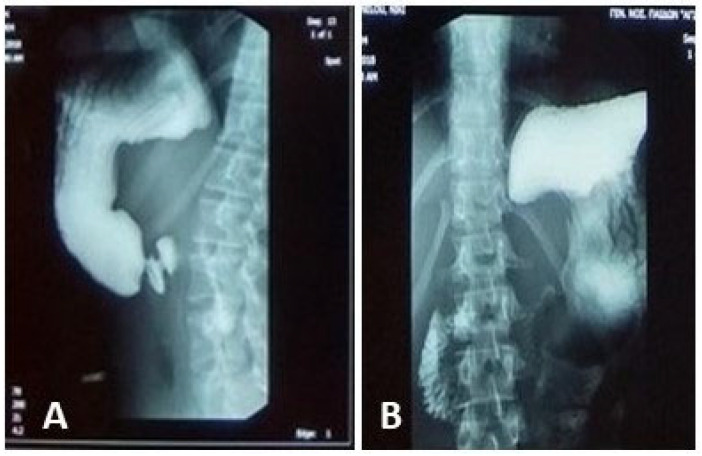
Upper GI contrast study: incomplete fixed stenosis due to an apparent extrinsic pressure effect on the second portion of the duodenum. (**A**): Delayed progression of contrast material into the duodenum; (**B**): Gastric cascade configuration.

An upper GI contrast study demonstrated an incomplete fixed stenosis due to an apparent extrinsic pressure effect on the second portion of the duodenum, which impeded the passage of contrast medium. No intrinsic strictures or obstructive lesions were identified. Additional findings included antral dyskinesia and antiperistalsis, evidenced by delayed passage of contrast material into the duodenal bulb. A folding of the gastric fundus was also noted Figure 1.

An initial abdominal ultrasound revealed no pathological findings. The major abdominal vessels, including the abdominal aorta, inferior vena cava, celiac trunk, renal, and iliac arteries, demonstrated normal patency. However, the aortomesenteric angle was measured at 20–22°, significantly reduced from the normal range (38–56°). The aortomesenteric distance was 8 mm—technically within the lower limit of normal, but considered pathological in the clinical context.

A Doppler ultrasound (U/S triplex) of the mesenteric vessels and abdominal aorta provided further detail. In the sagittal view, the angle between the aorta and the superior mesenteric artery (SMA) could not be measured due to the overlapping origin of the vessels. In the transverse view, the aortomesenteric distance was found to be only 3–4 mm, a clearly pathological finding. Additionally, the superior mesenteric vein (SMV) appeared to be abnormally positioned to the left of the SMA. The head of the pancreas could not be clearly visualized.

Subsequent MRI of the upper and lower abdomen and MR enterography revealed no abnormal findings.

**Figure 2:**
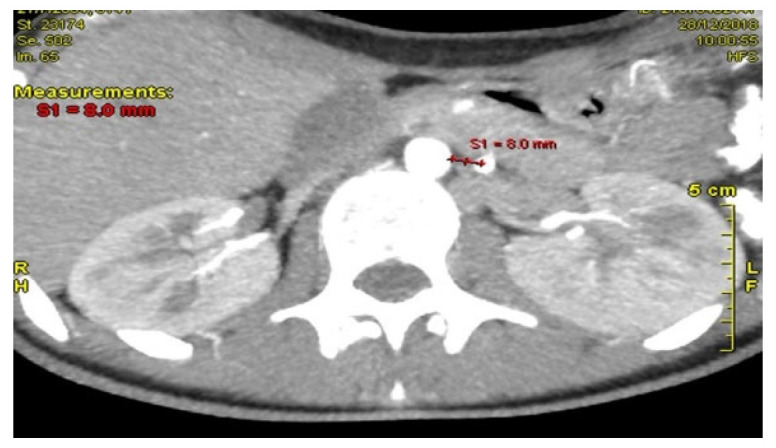
CTA: The aortomesenteric angle was significantly reduced to 6°.

**Figure 3:**
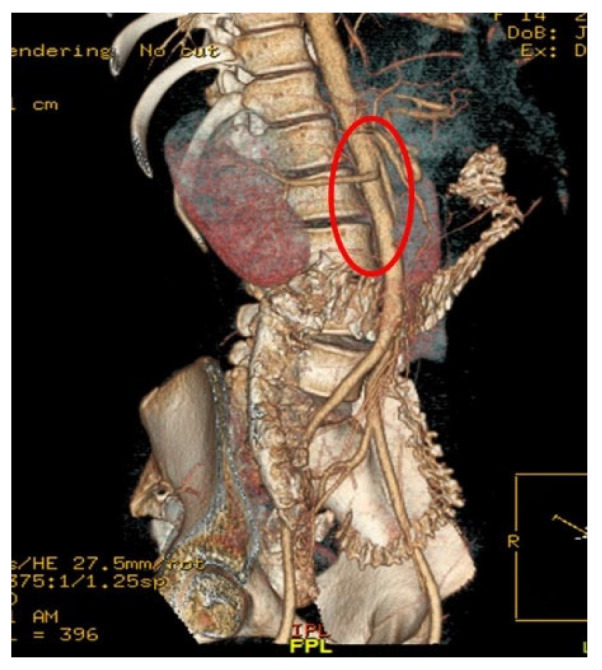
CTA: The aortomesenteric distance measured 4 mm.

**Figure 4:**
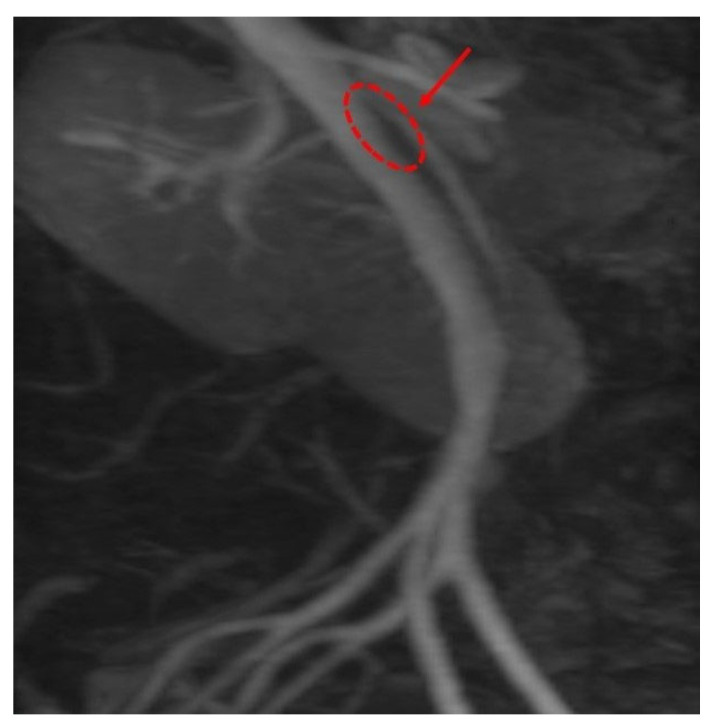
CTA: Findings consistent with superior mesenteric artery SMAS.

Computed Tomography Angiography (CTA) Figure 2, provided definitive anatomical correlation. At the level where the third portion of the duodenum crosses between the abdominal aorta and the SMA, the aortomesenteric angle was significantly reduced to 6° (normal: 38–56°), Figure 3 and the aortomesenteric distance measured 4 mm (normal: >10 mm, with <8 mm considered pathological). Figure 4 These findings were consistent with SMAS, a rare condition caused by vascular compression of the third portion of the duodenum between the SMA and the aorta.

### 2.4. Management and Outcome

Initially, a nasogastric tube was placed, resulting in some improvement. However, after a few days, she began vomiting again, was unable to tolerate even continuous feeding, and continued to lose weight. Consequently, the patient was managed conservatively with central parenteral nutrition for 8 weeks. During this period, she demonstrated clinical improvement and gained 5 kg, reaching a body weight of 51.4 kg. Due to persistent anatomical findings and failure of conservative therapy to restore normal duodenal patency, surgical intervention was performed. The STRONG (Strong’s procedure) was performed in our patient. It involves division of the ligament of Treitz and repositioning of the duodenum to reduce the extrinsic vascular compression. At seven months postoperatively, the patient remained symptom-free, with a total weight gain of 11 kg since initial presentation, with an improvement in her body weight 58 kg and BMI to 20.7. Her nutritional status and gastrointestinal function had normalised, with no recurrence of vomiting or abdominal pain.

## 3. Discussion

The present case highlights a classic yet often-underrecognized scenario of SMAS—an adolescent with significant weight loss, persistent vomiting and epigastric pain, and imaging evidence of marked reduction in aortomesenteric angle and distance. Several key issues merit discussion: pathophysiology, diagnosis and imaging, conservative versus surgical management, and special considerations in adolescents.

### 3.1. Pathophysiology and Risk Factors

SMAS results from compression of the third portion of the duodenum between the Superior Mesenteric Artery (SMA) and the Abdominal Aorta, typically when the intervening fat pad is depleted and the aortomesenteric angle narrows (often <25–30°) and the distance reduces (often <8–10 mm) [1,2]. Common precipitants include rapid weight loss, catabolic states, prolonged bed rest, scoliosis correction or other surgery, and psychiatric eating disorders. In one systematic review of SMAS post-scoliosis surgery (mean age ~15.8 years, mean BMI ~16.5) the authors emphasized the adolescent population with low BMI as being high-risk [3].

In our patient, the 7 kg weight loss over six months (BMI ~16) likely led to fat pad depletion and thus narrowed the angle/distance (6° angle, 4 mm distance), consistent with the mechanism of duodenal compression.

### 3.2. Clinical Presentation and Diagnostic Challenges

SMAS often presents with non-specific upper gastrointestinal (GI) symptoms: postprandial epigastric pain, nausea, recurrent vomiting, early satiety, bloating—all of which may mimic more common GI pathologies [2].

In adolescents, this can be even more difficult to diagnose, as features overlap those of eating disorders, functional GI disorders or other obstructive/neurological causes. For instance, one review of SMAS in anorexia nervosa emphasizes the challenge of distinguishing symptoms of the eating disorder from those of duodenal compression [4].

Across recent pediatric reports from the last five years of (SMAS, most patients were adolescents, with a smaller proportion of younger children affected. Clinical presentation was dominated by chronic or recurrent abdominal pain, vomiting, and weight loss, frequently with delayed diagnosis, and one case reported an association with IgA vasculitis. Radiologic confirmation of duodenal compression was central to diagnosis in all cases. Initial conservative management, including nutritional support and gastrointestinal decompression, was attempted when feasible and was successful in a minority of patients, particularly those with a shorter duration of symptoms. However, most patients ultimately required surgical intervention—most commonly laparoscopic duodenojejunostomy—which was associated with high rates of symptom resolution, favorable outcomes, minimal long-term morbidity, and rare postoperative complications [5,6,7,8,9,10]. The details of pediatric case series and case reports published within the last five years are summarized in Table 1.

In our case, the work-up included normal endoscopy/histology, raised calprotectin (which complicated the picture and initially steered toward IBD), and a neurologic event (blurred vision/numbness) causing further diagnostic uncertainty. This underscores the point that SMAS is a diagnosis of exclusion and requires high clinical suspicion.

This case differs from most SMAS reports in several ways. Unlike many pediatric cases triggered by scoliosis surgery or rapid postoperative weight loss, our patient developed SMAS gradually, without surgical risk factors, and with non-specific gastrointestinal symptoms. The elevated fecal calprotectin and strong family history of IBD—features rarely described in SMAS—initially suggested an inflammatory pathology and contributed to diagnostic delay. The transient neurological episode further complicated the presentation. Additionally, the patient showed unusually severe anatomical compression (aortomesenteric angle 6°, distance 4 mm) despite the absence of structural precipitating factors. Her excellent postoperative recovery and substantial weight gain contrast with reports describing longer-term nutritional dependence in some patients.

### 3.3. Imaging and Diagnostic Criteria

Imaging is central to diagnosis. Typical modalities include upper GI contrast studies (showing delayed contrast passage or duodenal dilatation with obstruction at the third part of the duodenum) and cross-sectional imaging (CT or MR angiography) showing reduced aortomesenteric angle and/or distance [11].

Recent literature emphasises that point-of-care ultrasound (POCUS) may assist initial screening (e.g., measuring aortomesenteric distance) though its sensitivity/specificity remains limited. In our patient, the CT angiography with angle of 6° and distance 4 mm provided a clear anatomical correlate, consistent with published pathologic thresholds (angle often <25°, distance <8–10 mm) [12].

It is worth emphasizing that imaging findings must be interpreted in the clinical context—many individuals may have reduced aortomesenteric angle without symptoms; thus, correlation with gastrointestinal obstruction symptoms is key.

### 3.4. Management: Conservative Versus Surgical

Current consensus suggests that conservative management should be the first-line approach in stable patients, especially those with weight loss as the precipitant. Weight restoration (via high-calorie diet, enteral or parenteral nutrition), bowel decompression (nasogastric tube), fluid/electrolyte correction, and postural/feeding modifications (e.g., left lateral/prone positioning) are recommended [13]. A suggested Stepwise Management Approach for Pediatric SMAS appears in Table 2.

For instance, studies emphasize that weight restoration remains the primary goal and that concomitant psychiatric/behavioural factors (especially in eating-disorder patients) must be addressed [2,13].

However, when conservative measures fail (persistent vomiting/obstruction, nutritional deterioration, inability to restore weight, or ongoing anatomical compression), surgical intervention is indicated. Surgical options include duodenojejunostomy (open or laparoscopic) [14,15], gastrojejunostomy, Strong’s procedure (division of the ligament of Treitz). A systematic review found that for SMAS the failure-to-treat rate after surgery was ~21% (i.e., persistent symptoms) and that careful pre-operative selection is important [14].

More recently, a meta-analysis in 2024 comparing duodenojejunostomy versus Roux-en-Y gastrojejunostomy found pooled efficacy of duodenojejunostomy ~0.84 (95% CI 0.74–0.90) and safety ~0.89 (95% CI 0.81–0.94) in SMAS [16].

In our case, after ~8 weeks of parenteral nutrition and modest weight gain, the decision to proceed to surgery was appropriate given persistent anatomical compression and incomplete response. The favourable outcome at 7-month follow-up (11 kg weight gain, symptom-free) aligns with published outcomes of surgical management in the adolescent/young adult population.

### 3.5. Special Considerations in Adolescents and Diagnostic Delays

Adolescents with SMAS may present in the context of weight loss from eating disorders, scoliosis correction, or other catabolic states. The finding in the systematic review of SMAS after scoliosis surgery underscores that adolescents are vulnerable [3].

In our patient, earlier suspicion of SMAS—given the significant weight loss and recurrent vomiting with epigastric pain—might have shortened the diagnostic delay. The diagnostic process was further complicated by additional findings, including elevated fecal calprotectin and a family history of inflammatory bowel disease (IBD), which initially suggested alternative gastrointestinal pathology.

Persistent vomiting and weight loss in an adolescent should prompt consideration of SMAS (among other causes) once more common etiologies have been excluded. In addition, interdisciplinary collaboration (gastroenterology, nutrition, surgery, psychiatry) is beneficial, particularly if an eating-behaviour component is present.

### 3.6. Lessons from This Case and Implications for Practice

Early measurement of aortomesenteric angle/distance (via CT/MR or dedicated imaging) in adolescents with weight loss + upper GI obstructive symptoms can shorten time to diagnosis. Conservative nutrition-first strategies remain cornerstone but require close monitoring; if no improvement within ~2–4 weeks (as most literature suggests), surgical referral should be considered [15,17]. Because surgical outcomes are generally good (but not perfect) and failure rate ~20–25% in selected series, preoperative counselling and postoperative follow-up are essential [8]. The interplay of other GI/inflammatory findings (e.g., raised fecal calprotectin) may distract from the primary mechanical problem; hence SMAS remains a diagnosis of exclusion and requires multidisciplinary evaluation. Nutritional rehabilitation in adolescents must account for ongoing growth and development; the weight gain of 11 kg in 7 months in your case is an excellent outcome and likely contributed to durable resolution.

This case highlights that SMAS should remain in the differential diagnosis even when inflammatory markers or family history point toward IBD. Early cross-sectional imaging is essential when symptoms persist despite normal endoscopy. The use of prolonged parenteral nutrition as initial therapy—followed by timely surgery when obstruction persisted—illustrates an effective stepwise approach. Finally, the multidisciplinary coordination across gastroenterology, neurology, radiology, nutrition, and surgery was crucial in resolving a misleading and atypical presentation.

### 3.7. Limitations and Future Directions

The literature on SMAS remains limited by its rarity: many reports are case series or case reports. There is no large-scale prospective study in adolescents. Future research should address: (1) thresholds of aortomesenteric angle/distance that predict symptomatic obstruction; (2) optimal duration of conservative therapy before surgery; (3) long-term outcomes of adolescents who undergo surgery (growth, nutritional status, quality of life); (4) the role of novel imaging modalities (ultrasound, dynamic MRI) and minimally invasive surgical approaches (laparoscopic duodenojejunostomy, robotic surgery). Additionally, the psychological and behavioural context (especially in adolescents with disordered eating) is critical and should be incorporated into management algorithms.

## 4. Conclusions

Superior Mesenteric Artery syndrome (SMAS), though rare, should be considered in adolescents presenting with persistent upper gastrointestinal symptoms and significant weight loss, especially when common causes have been excluded. This case highlights the diagnostic complexity when non-specific symptoms are accompanied by misleading findings such as elevated fecal calprotectin and a family history of inflammatory bowel disease. Early recognition through targeted imaging—particularly measurement of the aortomesenteric angle and distance—is crucial for timely diagnosis. While conservative management remains first-line, surgical intervention may be necessary in cases with persistent obstruction. Multidisciplinary collaboration and close nutritional follow-up are essential to ensure full recovery and long-term wellbeing in affected adolescents.

## Figures and Tables

**Table 1:** Case reports and case series of pediatric patients with SMAS reported in the last five years.

Study Type	Age Range	Key Clinical Features	Management	Outcomes
Case series (*N* = 8) [10]	Adolescents (median ~16 yrs)	Chronic abdominal pain, vomiting, weight loss; multiple comorbidities; radiologic SMAS	Laparoscopic duodenojejunostomy (after failed conservative therapy)	Most had symptom resolution; one postop SBO; no long-term need for supplemental nutrition
Case report [5]	8 yrs	Chronic abdominal pain and vomiting since early childhood	Surgical duodenojejunostomy	Complete symptom resolution
Case report [6]	11 yrs	Abdominal pain, intermittent vomiting, short symptom duration	Conservative (nutritional support, decompression)	Clinical improvement without surgery
Case report [7]	12 yrs	Acute persistent vomiting, abdominal pain	Surgery after failed conservative therapy	Symptom resolution
Case report [8]	13 yrs	SMAS associated with IgA vasculitis (HSP), bilious vomiting	Surgical management	Clinical improvement
Case report [9]	10 yrs	Chronic abdominal pain, duodenal obstruction	Surgical bypass	Good recovery

**Table 2:** Suggested Stepwise Management Approach for Pediatric SMAS.

Step	Intervention	Indication/Notes
1	Initial conservative measures	Nutritional support (oral/enteral if tolerated), NG decompression
2	Nutritional optimization	TPN if oral/enteral intake insufficient; monitor for weight gain and symptomatic improvement
3	Repeat dynamic imaging	Assess aortomesenteric angle/distance and persistence of duodenal compression
4	Evaluate response to therapy	If symptoms persist despite weight gain and improved nutrition
5	Surgical intervention	Indicated for refractory cases or ongoing TPN dependence despite adequate conservative management
6	Post-operative rehabilitation	Gradual reintroduction of feeding, nutritional follow-up, symptom monitoring

## Data Availability

The original contributions presented in this study are included in the article. Further inquiries can be directed to the corresponding author.

## Abbreviations

The following abbreviations are used in this manuscript:

SMA	Superior mesenteric artery
SMAS	Superior mesenteric artery syndrome
BMI	Body mass index
GCS	Glasgow Coma Scale
CT	Computed Tomography
GI	Gastrointestinal
U/S	Ultrasound
SMV	Superior mesenteric vein
MRI	Magnetic Resonance imaging
MRA	Magnetic resonance angiography
